# Rethinking Reallocations: Conceptual Limits of the Market Activity Index as a Measure of Competition and Purchasing: Comment on "Measuring Active Purchasing in Healthcare: Analyzing Reallocations of Funds Between Providers to Evaluate Purchasing Systems Performance in the Netherlands"

**DOI:** 10.34172/ijhpm.9322

**Published:** 2025-11-22

**Authors:** Misja C. Mikkers

**Affiliations:** ^1^Health Technology and Services Research (HTSR), Faculty of Behavioural, Management and Social Sciences (BMS), University of Twente, Enschede, The Netherlands.; ^2^Centre for Healthcare Operations Improvement and Research, University of Twente, Enschede, The Netherlands.

**Keywords:** Healthcare Financing, Selective Contracting, Competition in Healthcare Markets, Provider Networks

## Abstract

Stadhouders et al^1^ introduce the Market Activity Index (MAI) to assess the performance of healthcare systems, concluding that low budget reallocations in the hospital sector cast doubt on the effectiveness of managed competition and purchasing. We argue that while the MAI is a valuable descriptive tool, its interpretation as a proxy for competition is conceptually problematic. The index captures realized revenue flows, which may result from patient mobility, exogenous shocks, or administrative changes, rather than insurer behavior. Furthermore, selective contracting may be used for objectives such as risk selection rather than provider efficiency, particularly in segments of the market with low utilization. Without a normative benchmark or ability to disentangle strategic from structural effects, the MAI risks conflating system dynamics with market failure. We conclude that the MAI is best viewed as a measure of budgetary volatility, not a standalone indicator of competitive intensity or purchaser effectiveness.

## Introduction

 The Market Activity Index (MAI) introduced by Stadhouders et al,^1^ which introduces the MAI is a novel attempt to quantify budget reallocations in healthcare markets. By analyzing reallocations of provider revenues across sectors—including hospitals, long-term care, and municipally procured services—the authors aim to test whether competitive purchasing arrangements, particularly managed competition in the hospital sector, lead to greater shifts in market shares, as predicted by theory. Their central finding is that the MAI is relatively low in the hospital sector, and not significantly higher than in the non-competitive long-term care sector. This, combined with regression results showing no clear link between selective contracting, provider quality, and revenue changes, leads the authors to question whether managed competition and active purchasing have achieved their intended allocative effects.

 While the paper provides an important contribution by developing an accessible and policy-relevant performance metric, this commentary argues that the interpretation of its findings requires caution. Specifically, we identify two methodological concerns. First, the MAI, although novel, suffers from conceptual ambiguities that limit its ability to distinguish between competition, purchasing activity, and broader system dynamics. Second, the regression analysis, while carefully executed, is built on strong assumptions about the relationship between contracting, quality, and expenditure shifts—assumptions that may not hold in practice. Importantly, we do not dispute the authors’ broader conclusion that the hospital sector in the Netherlands shows limited evidence of active purchasing or meaningful competition. Rather, our commentary focuses on the limitations of the MAI as a performance measure—particularly its ambiguity and the risk of misinterpreting what low or high values actually signal.

## Rethinking What Market Activity Index Measures

 The MAI introduced by Stadhouders et al^1^ is a novel attempt to quantify reallocations in healthcare markets, intended as a proxy for active purchasing and a reflection of the competitive functioning of managed care. However, the interpretation of the MAI in the paper tends to conflate conceptually distinct elements: competition, active purchasing, and market share dynamics. In particular, the MAI bears a strong resemblance to Elzinga-Hogarty-style approaches (EH-approach) to market definition, which use patient flows to delineate market boundaries. Both approaches treat observed movement—whether of patients or revenue—as evidence of competitive interaction. Yet, as Frech et al^2^ argue, patient flows may occur for reasons entirely unrelated to price sensitivity or competitive pressure: patients may travel for specialized care, personal convenience, or referrals, not in response to competition. In this sense, both MAI and EH-type metrics suffer from the same core limitation: they reflect realized outcomes, not competitive constraints.

 One key difference lies in the data. Whereas the EH-approach requires detailed individual-level patient data to track flows across geographic markets, the MAI uses publicly available aggregated financial data to approximate similar patterns at the level of provider expenditures. In that sense, the MAI can be viewed as a kind of “aggregate EH approach,” using revenue shifts as a proxy for patient movement. While this makes the measure accessible and scalable, it inherits the interpretive weaknesses of its micro-level counterpart—particularly the inability to distinguish between strategic and exogenous sources of change.

 Moreover, the theoretical foundations of competitive insurance markets suggest that under perfect competition, insurers will contract with all providers to maximize enrollee utility (See eg, Appendix 2 of Capps et al^3^). If prices are fixed and all providers are similarly efficient, no reallocations are needed or expected—meaning the MAI may be low precisely because the market functions well. Conversely, a low MAI may also emerge in uncompetitive markets with limited provider choice, where purchasers lack the tools or leverage to steer funds. High MAI values are similarly ambiguous: they may reflect dynamic purchasing in the face of heterogeneity, or simply volatility driven by exogenous shocks. The authors themselves acknowledge this complexity, noting that “the optimal MAI is likely dependent on market characteristics,” and that high volatility may carry costs. Nevertheless, the empirical analysis in the paper largely treats low MAI as evidence against the effectiveness of managed competition. Without a normative benchmark or a method to decompose MAI into its strategic and non-strategic components, the index remains an ambiguous indicator. It may reveal budget volatility, but not necessarily market failure and certainly not the presence or absence of meaningful competition.

 Even when MAI is interpreted not as a proxy for competition but for active purchasing, important limitations remain.

 Strategic or active purchasing implies that third-party payers—such as insurers, regional care offices, or municipalities—use their purchasing power to steer resources toward more efficient or higher-quality providers. However, the MAI only captures changes in provider revenue over time, without evidence on whether those changes are the result of deliberate, selective contracting decisions. Reallocations may occur due to structural factors outside the control of purchasers, such as changing patient needs, regional population shifts, mergers, or administrative changes to accounting or reporting practices. Moreover, some forms of active purchasing—such as quality improvement within a fixed budget, negotiated price reductions, or care substitution—may leave overall revenue allocations unchanged. In these cases, the MAI would remain flat even if purchasers were highly active and effective. Conversely, a high MAI may reflect turbulence rather than purposeful reallocation: patient churn, provider instability, or policy reform-induced volatility.

 The regression analysis presented in the paper attempts to address this by linking market share changes to a measure of selective contracting and to provider quality. Yet no significant relationships are found, suggesting that changes in provider revenue are not systematically related to either contracting behavior or observable quality metrics. While the authors rightly conclude that this weakens the case for active purchasing in the Dutch hospital market, it also raises concerns about the validity of the MAI itself as a behavioral proxy. If the MAI is uncorrelated with selective contracting and with quality—the two main channels through which active purchasing is expected to operate—then it is unclear whether the index is capturing anything meaningful about purchaser strategy.

 Furthermore, the assumption that selective contracting is primarily used to reward efficiency may not hold. As Bijlsma et al^4^ show, insurers may also use selective contracting to attract favorable risk types, even under perfect risk adjustment. Specifically, plans that offer narrow provider networks often appeal to younger, healthier enrollees who are more likely to switch and thus more responsive to price differences. These individuals tend to have low healthcare utilization, especially in the hospital sector, and may never realize their covered care. For these enrollees, insurers need not adjust their contracting patterns, as most contracting occurs through their broad plans that include nearly all providers. In this sense, narrow networks serve mainly as a demand-side selection tool rather than a supply-side purchasing strategy. As a result, insurers engaging in selective contracting for demand-side positioning may not induce any meaningful reallocation of hospital revenues. In this scenario, selective contracting affects who enrolls, not where money flows, further weakening the link between selective purchasing and the MAI. A low MAI, then, does not necessarily indicate a lack of insurer activity—it may simply reflect that selective networks are strategically deployed in segments of the market where hospital spending is minimal. A low MAI, then, does not necessarily indicate a lack of insurer activity—it may simply reflect that selective contracting operates in a segment of the market where spending is low, and where the primary objective is enrollee selection, not provider steering.

## Concluding Reflections

 The MAI proposed by Stadhouders et al^1^ offers a creative and data-driven attempt to operationalize an important policy question: whether purchasing systems reallocate funds in ways that improve efficiency. As a cross sectoral tool, the MAI is appealing for its simplicity and potential to inform policy debates. However, as this commentary has argued, the interpretation of the MAI as evidence of competition or active purchasing is problematic. The index conflates multiple mechanisms—from patient mobility to policy-induced shocks—and cannot distinguish strategic insurer behavior from passive system dynamics. Moreover, the regression analysis does not establish a robust link between selective contracting, quality, and budgetary reallocations. In some cases, selective contracting may primarily serve demand-side strategies such as risk selection, particularly in low-utilization populations, without affecting provider revenue at all.

 None of this is to suggest that the MAI is without value—but rather that it should be treated as a descriptive measure of budget volatility, not a standalone indicator of market performance or purchaser effectiveness. Future research could strengthen its interpretive value by combining MAI trends with granular data on contracting decisions, patient flows, or price negotiations. Alternatively, more direct measures of purchaser behavior, such as changes in service scope, payment terms, or performance-linked incentives, may better capture what active purchasing entails in practice. The question of how to monitor and evaluate purchasing performance remains essential—but the metrics we use must be carefully aligned with the mechanisms we seek to understand.

## Disclosure of artificial intelligence (AI) use

 Not applicable.

## Ethical issues

 Not applicable.

## Conflicts of interest

 Author declares that he has no conflicts of interest.

## References

[1] Stadhouders NW, Koolman X, Tanke MA, Maarse H, Jeurissen PP (2023). Measuring active purchasing in healthcare: analysing reallocations of funds between providers to evaluate purchasing systems performance in the Netherlands. Int J Health Policy Manag.

[2] Frech HE III, Langenfeld J, McCluer RF (2003). Elzinga-Hogarty tests and alternative approaches for market share calculations in hospital markets. Antitrust Law J.

[3] Capps C, Dranove D, Satterthwaite M (2003). Competition and market power in option demand markets. Rand J Econ.

[4] Bijlsma M, Boone J, Zwart G (2014). Competition leverage: how the demand side-affects optimal risk adjustment. Rand J Econ.

